# Patent and Trade Marks

**Published:** 1882-03-20

**Authors:** 


Patent and Trade Marks.—Attention is invited to the advertisement of Messrs. Parke, Davis & Co., in relation to the injury threatened public, professional and scientific interests by the abuse of the law relating to patents and trade marks. The advertisement will be found interesting and instructive. Messrs. Parke, Davis & Co. not only defend their house against assaults unjustly made, but define intelligibly what is meant by trade marks, and the true object and intent of the law relating to the same.

